# Cooper-pair distribution function $D_{cp}(\omega ,T_c)$ for superconducting $\hbox {D}_3\hbox {S}$ and $\hbox {H}_3\hbox {S}$

**DOI:** 10.1038/s41598-021-02081-w

**Published:** 2021-11-19

**Authors:** G. I. González-Pedreros, J. A. Camargo-Martínez, F. Mesa

**Affiliations:** 1 Faculty of Natural Sciences, Universidad del Rosario, Carrera 24 # 63C - 69, 111221 Bogotá, DC Colombia; 2 Grupo de Investigación en Ciencias Básicas, Aplicación e Innovación - CIBAIN, Universidad Internacional del Trópico Americano - Unitrópico, Yopal, Casanare Colombia

**Keywords:** Superconducting properties and materials, Condensed-matter physics

## Abstract

Cooper-pair distribution function, $D_{cp}(\omega ,T_c)$, is a recent theoretical proposal that reveals information about the superconductor state through the determination of the spectral regions where Cooper pairs are formed. This is built from the well-established Eliashberg spectral function and phonon density of states, calculated by first-principles. From this function is possible to obtain the $N_{cp}$ parameter, which is proportional to the total number of Cooper pairs formed at a critical temperature $T_c$. Herein, we reported $D_{cp}(\omega ,T_c)$ function of the compressed $D_3S$ and $H_3S$ high-$T_c$ conventional superconductors, including the effect of stable sulfur isotopes in $H_3S$. $D_{cp}(\omega ,T_c)$ suggests that the vibration energy range of 10–70 meV is where the Cooper pairs are possible for these superconductors, pointing out the possible importance of the low-energy region on the electron–phonon superconductivity. This has been confirmed by the fact that a simple variation in the low-frequency region induced for the substitution of S atoms in $H_3S$ by its stable isotopes can lead to important changes in $T_c$. The results also show proportionality between $N_{cp}$ parameter and experimental or theoretical $T_c$ values.

## Introduction

The Bardeen–Cooper–Schrieffer theory (BCS) provides a way for achieving high superconducting transition temperature $\hbox {T}_c$; to reach an appropriate combination between high-frequency phonons, strong electron–phonon coupling, and a high density of states^1^. This condition could be fulfilled for metallic hydrogen and covalent compounds dominated by hydrogen^2,3^, due to the fact that hydrogen atoms provide the necessary high-frequency phonon modes as well as the strong electron-phonon coupling^4^. Ashcroft and Richardson^5^ reported the possibility of superconductivity in a dense phase of hydrogen, which becomes metallic while retaining diatomic character. Metallic hydrogen is a candidate to report a high critical temperature $\hbox {T}_c$^3^, but it has not been obtained yet. However, several experimental and theoretical works have explored compounds where hydrogen is the main component^6–10^. Two of these compounds are $\hbox {D}_3\hbox {S}$ and $\hbox {H}_3\hbox {S}$, of which experimental measurements showed $T_c$ values of 150 and 203 K^4^, respectively.

In a previous theoretical work, Cooper-pair distribution function $D_{cp}(\omega ,T_c)$ was reported^10^. This is a function built from the well-established Eliashberg spectral function and phonon density of states, which reveals information about the superconductor state through the determination of the spectral regions for Cooper pairs formation^11^. The $D_{cp}(\omega ,T_c)$ of compressed $\hbox {H}_3\hbox {S}$ revealed that the low-frequency vibration region is where Cooper pairs are possible^10^. For bcc Niobium this function managed to simulate the $\hbox {T}_c$ Nb anomalies^11^ measured at 5 and 50 GPa. The physical implications of $D_{cp}(\omega ,T_c)$ function deserve to be evaluated in more detail.

On the other hand, it is well known that isotope mass has a functional dependence on vibrational states in any crystal structure. Each term in the dynamical matrix depends of atom-masses, $m_i$ and $m_j$, by $1/\sqrt{m_im_j}$^12^. In conventional superconductors, it is natural to expect a relationship between $\hbox {T}_c$ and the vibrational states, then one might expect as isotope mass increases (or decreases) the corresponding $\hbox {T}_c$ will weaken (or improve). Previous experimental and theoretical studies showed that the anomalous sulfur-derived superconducting isotope effect is evidence of the existence of phonon-mediated pairing mechanism of superconductivity in $\hbox {D}_3\hbox {S}$ and $\hbox {H}_3\hbox {S}$ superconductors^4,13^. This fact validates the calculation of $D_{cp}(\omega ,T_c)$ function of these superconductors.

In this paper, we present the analysis of Cooper-pair distribution function $D_{cp}(\omega ,T_c)$ of compressed $\hbox {D}_3\hbox {S}$ and $\hbox {H}_3\hbox {S}$ high-$\hbox {T}_c$ conventional superconductors, including the effect of all stable sulfur isotopes in $\hbox {H}_3\hbox {S}$.

### Cooper-pair distribution function $D_{cp}(\omega ,T_c)$

Conventional superconductivity is mediated by Cooper pairs. These are possible if a set of specific physical conditions are satisfied. We can associate a probability of occurrence of each of these^9–11^ through occupied and vacant electronic states, vibration energy states, electron-phonon interaction, Fermi–Dirac and Bose–Einstein distributions. Thus, simultaneous likelihood summed over all electronic states defines the Cooper-pair distribution function, $D_{cp}(\omega ,T_c)$, that establishes the spectral range where Cooper pairs could be formed. $D_{cp}(\omega ,T_c)$ function is given by

1 $$\begin{aligned} D_{cp}(\omega ,T_c)=\int _{E_F-\omega _s}^{E_F+\omega _s}\int _{E_F-\omega _s}^{E_F+\omega _s} g_{ep}^s (\epsilon ,\omega ,T_c)\times g_{ep}^b (\epsilon '+\omega ,\omega ,T_c)\times \alpha ^2(\omega )d\epsilon d\epsilon '. \end{aligned}$$

Here, $g_{ep}^s (\epsilon ,\omega ,T_c) \times g_{ep}^b(\epsilon '+\omega ,\omega ,T_c)$ is the probability at $\hbox {T}_c$ that: (i) one electron is in energy state $\epsilon$, a second one is in energy state $\epsilon ' + \omega$, (ii) two empty electronic energy states $\epsilon + \omega$ and $\epsilon '$, (iii) two electrons are coupled to a phonon with energy $\omega$, (iv) a vibrational energy state $\omega$, (v) an additional vibrational energy state $\omega$, and vi) the electrons coupling with a phonon, $\alpha ^2(\omega )$. The calculation contains the contribution of all the electrons in the energy interval $\pm \omega _s$ around the Fermi level ($E_F\pm \omega _s$). For more details, see Ref.^14^.

Furthermore, from $D_{cp}(\omega ,T_c)$ is possible get an estimate of the total number of Cooper pairs formed at temperature $T_c$ through a quantity proportional to it, $N_{cp}$ parameter,

2 $$\begin{aligned} N_{cp}=\int ^{\omega _{cut-off}}_{0}D_{cp}(\omega ,T_c)d\omega , \end{aligned}$$

where $\omega$ is a phonon energy and $\omega _{cut-off}$ is a cut-off energy so that to $\omega >\omega _{cut-off}$ the $D_{cp}(\omega ,T_c)$ is negligible.

## Method of calculation

In order to determine Cooper-pair distribution function $D_{cp}(\omega ,T_c)$, we require electronic density states, vibrational density states and Eliashberg function. To obtain these spectra from ab initio calculations, we first relax the internal degrees of freedom and the lattice vectors of the $Im\bar{3}m$ structure using the Broyden–Fletcher–Goldfarb–Shanno (BFGS) quasi-Newton algorithm^15^ at each pressure. From these relaxed structure configurations, we calculated electronic and phonon band structures, electron (DOS) and phonon (PhDOS) densities of states, and Eliashberg function $\alpha ^2F(\omega )$. We used a kinetic energy cut-off of 70 *Ry* for the expansion of the wave function into plane waves and 280 *Ry* for the density. To integrate over the Brillouin zone (BZ) we used for the electronic integration a *k*-grid of $32 \times 32 \times 32$ and for the phononic integration a *q*-grid of $8\times 8\times 8$ according to the Monkhorst–Pack scheme^15^. We performed the calculations using the pseudopotential plane-wave (PW) method of Perdew et al.^16^, the generalized gradient approximation (GGA) and a Troullier and Martins^17^ norm-conserving pseudopotential. The energy convergence and precision of all presented results were controlled, thresholds on total energy and for self-consistency were taken $10^{-18}$ *Ry* and $-10^{-14}$ *Ry*, such that it does not present imaginary frequencies. The $Im\bar{3}m$ $\hbox {D}_3\hbox {S}$ and $\hbox {H}_3\hbox {S}$ structures were verified as stable structures and these did not show phase transitions in the pressure interval (180–220 GPa) in accordance with a previous work^10^ and other ones^18,19^. The cut-off vibrational frequencies and a grid were chosen big enough to obtain a good precision in phonon structure and $\alpha ^2F(\omega )$, calculated within the density-functional perturbation theory (DFPT) frame^20,21^. We used the Quantum Espresso code^22^ with *pbe-kjpaw_psl* pseudopotential for H and *pbe-n-kjpaw_psl* pseudopotential for S, in all these calculations. The pressure conditions were calculated in the range where the high-$\hbox {T}_c$ was measured for $\hbox {D}_3\hbox {S}$ (180–220 GPa) and $\hbox {H}_3\hbox {S}$ (180 GPa). The calculation parameters used in this work are similar to previous works^9,10^ and other one^13^.

The so-called Umklapp processes contribute to the thermal and electrical properties of solids, these are originated from the interaction between phonon–phonon and electron–phonon. In particular, the Umklapp phonons come from anharmonic terms. Some authors^23–25^ suggest that Umklapp phonons must be included in theoretical studies that evaluating phonon interaction effects at high-temperature conditions (like room-temperature for superconductors). However, the presence of umklapp process is mainly associated with the electrical resistivity^26–29^, which is measured in the normal state and not in the superconductor one. Our calculations were considered in the superconducting state, and Umklapp processes have not been included^13,30^.

## Results and discussion

### $\hbox {D}_3\hbox {S}$ and $D_{cp}(\omega ,T_c)$ function

**Figure 1:**
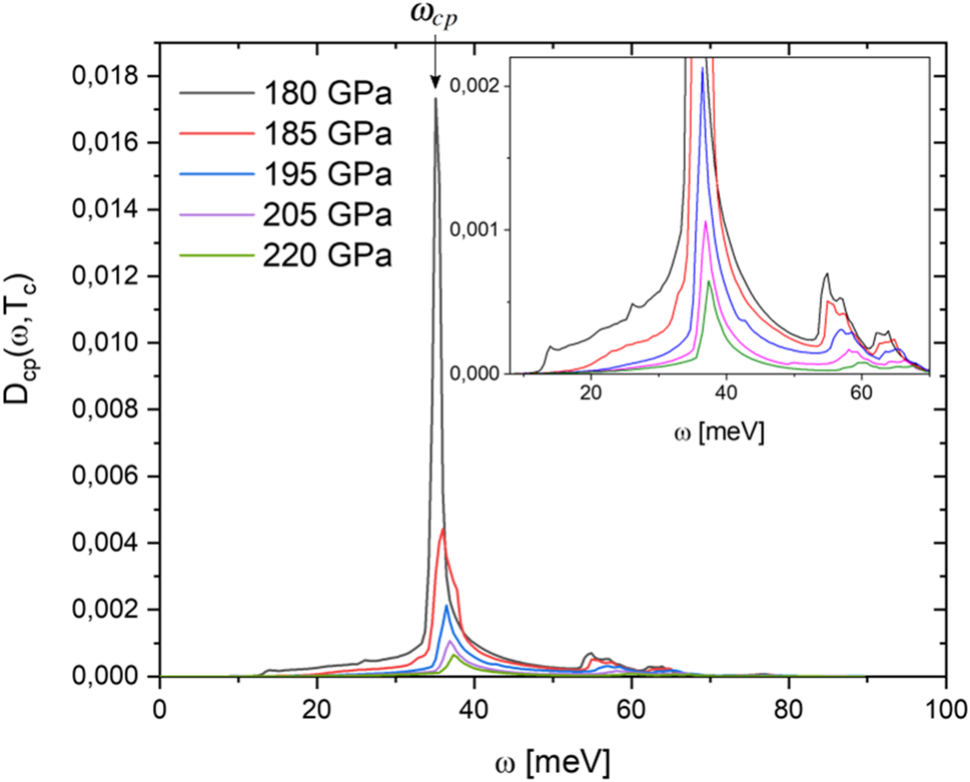
Cooper-pair distribution function $D_{cp}(\omega ,T_c)$ of $\hbox {D}_3\hbox {S}$ calculated at different pressures. The inset presents a zoom-in view of $D_{cp}(\omega ,T_c)$ from 10 to 70 meV.

Cooper-pair distribution functions $D_{cp}(\omega ,T_c)$ of $\hbox {D}_3\hbox {S}$ calculated at different pressures are shown in Fig. 1. It is observed that these functions have a shape that mimics a delta function centered around 35 meV. The $D_{cp}(\omega ,T_c)$ of $\hbox {D}_3\hbox {S}$ situates to Cooper pairs formation only in the 10–70 meV interval, pointing out the possible relevance of low-vibrational frequencies in the superconducting phenomenon.

These results indicate that it is only the low-frequency phonons (below 70 meV) that seem to contribute to the formation of Cooper pairs, which for $\hbox {D}_3\hbox {S}$ (and $\hbox {H}_3\hbox {S}$) come mainly from S atoms, with a lower contribution from the vibrational states of H atoms. It is important to note that for energies greater than 70 meV, where Cooper pairs seem not possible, there is no phononic contribution from S atoms, in these superconductors (see Ref.^13^). Furthermore, the $D_{cp}(\omega ,T_c)$ functions indicate that it is the phonon at 35 meV which mainly contributes to the Cooper pairs formation.

The importance and contribution of low-energy phonons in superconductivity have also been measured and reported. It is the case of the low-energy phonon (21 meV) $\hbox {A}_{{1g}}$ which seems to have a pivotal role in the superconductivity mechanism in ($\hbox {Li}_{1-x}\hbox {Fe}_x$)$\hbox {OHFe}_{1-y}$Se compound^31^. This $\hbox {A}_{{1g}}$ phonon comes from the Se atom. This study also reported a positive correlation between $\hbox {T}_c$ and the electron–phonon coupling (EPC) strength $\lambda _{A1g}$ for all categories of iron-based superconductors, which highlights the importance of low-energy phonons in conventional superconductors. The recent experimental studies of the electron–phonon interaction in superconductors have been made possible by the application of modern techniques such as Ultrafast optical spectroscopy, which in the future will allow a closer understanding of conventional superconductors (see Refs.^31–33^ and references therein).

**Table 1:** $\hbox {N}_{{cp}}$ and $\omega _{cp}$ values obtained from $D_{cp}(\omega ,T_c)$, and lattice parameter (a) calculated for $\hbox {D}_3\hbox {S}$ at different pressures, contrasted with their respective experimental $\hbox {T}_c$ values^4^.

Pressure [GPa]	a [Å]	$\hbox {T}_c$ [K]	$\omega _{cp}$ [meV]	$N_{cp}$ ($\times 10^{-3}$)
180	3.0162	150.39	35.67	42.21
190	3.0012	147.89	34.20	34.25
195	2.9939	146.64	35.10	17.22
200	2.9939	145.39	36.00	11.53
205	2.9798	144.14	36.45	8.89
210	2.9729	142.89	36.45	4.55
215	2.9662	141.64	36.90	5.77
220	2.9596	140.40	36.90	5.52

As it can be seen in Fig. 1, the increase in pressure induces a decrease in the area under $D_{cp}(\omega ,T_c)$, which means that the increase in pressure weakens the physical conditions (electron–phonon coupling) for Cooper pair formation. In this sense, Tian et al. suggest that the e–ph coupling can be weakened by anharmonic decay of the optical phonons into acoustic phonons^32^, which is an interesting proposal that could be evaluated in $\hbox {D}_3\hbox {S}$ (and $\hbox {H}_3\hbox {S}$). In Table 1 the values of characteristic peak ($\omega _{cp}$) and $\hbox {N}_{{cp}}$ parameters obtained from $D_{cp}(\omega ,T_c)$, and lattice parameters of $\hbox {D}_3\hbox {S}$ calculated at different pressures, compared with their respective experimental $\hbox {T}_c$ values are presented .

It is observed in Table 1 that the $\hbox {N}_{{cp}}$ values calculated are correlated with experimental $\hbox {T}_c$ values. An increase in $\hbox {N}_{{cp}}$ implies an increase in $\hbox {T}_c$, and both decrease appreciably with increasing pressure. It can also be observed that the $\omega _{cp}$ values have a behavior opposite. However, it is important to note that the variation in $\omega _{cp}$ is small, around 1 meV, despite the fact that the changes in pressure and $\hbox {T}_c$ are considerable ($\Delta P=40$ GPa and $\Delta T_c=10$ K). On the other hand, the lattice parameters of $\hbox {D}_3\hbox {S}$ obtained only show a slight compression (0.06 Å) induces by a pressure variation of 40 GPa, which confirms the strong stability of this structure in the pressure range of 180 to 220 GPa^34^.

### $\hbox {D}_3\hbox {S}$ and $\hbox {H}_3\hbox {S}$ comparison

**Figure 2:**
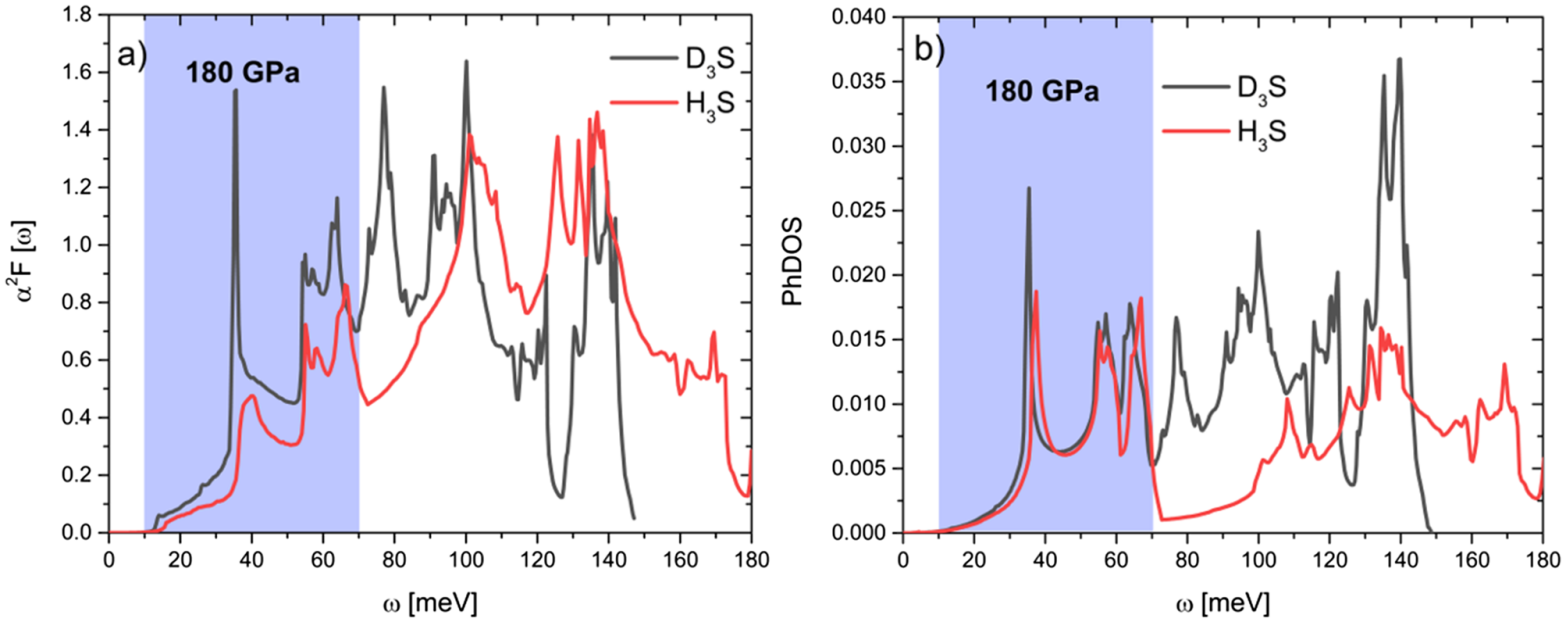
Comparison between (**a**) Eliashberg spectral functions $\alpha ^2F(\omega )$ and (**b**) phonon density of states (PhDOS) calculated at 180 GPa for $\hbox {D}_3\hbox {S}$ (black line) and $\hbox {H}_3\hbox {S}$ (red line). The highlighted area corresponds to the region where Cooper pairs occur.

Eliashberg spectral functions $\alpha ^2F(\omega )$ and Phonon density of states (PhDOS) calculated at 180 GPa for $\hbox {D}_3\hbox {S}$ and $\hbox {H}_3\hbox {S}$ are shown in Fig. 2.

In Fig. 2 it is observed that when the hydrogen is substituted for deuterium in $\hbox {H}_3\hbox {S}$, their corresponding $\alpha ^2F(\omega )$ and PhDOS functions are compressed along the energy-axis. This may be due mainly to the modification of the vibrational spectrum, generated by the change of atomic mass. Note in Fig. 2b that below 70 mev the PhDOS are almost identical, which implies that the contribution of S atoms to vibrational spectrum (see Fig. 4 in Ref.^13^) is not affected by the substitution of H by D, behaving as uncoupled atoms. The spectral differences observed between $\hbox {D}_3\hbox {S}$ and $\hbox {H}_3\hbox {S}$ (both in $\alpha ^2F(\omega )$ and PhDOS) contain physical information that leads to a difference around 25 K between their perspectives $\hbox {T}_c$, at 180 GPa. However, the direct analysis of these spectral functions does not allow for inferring with total certainty for which characteristics cause this behavior. The origin and difference of $T_c$ between these conventional superconductors remain hidden.

**Figure 3:**
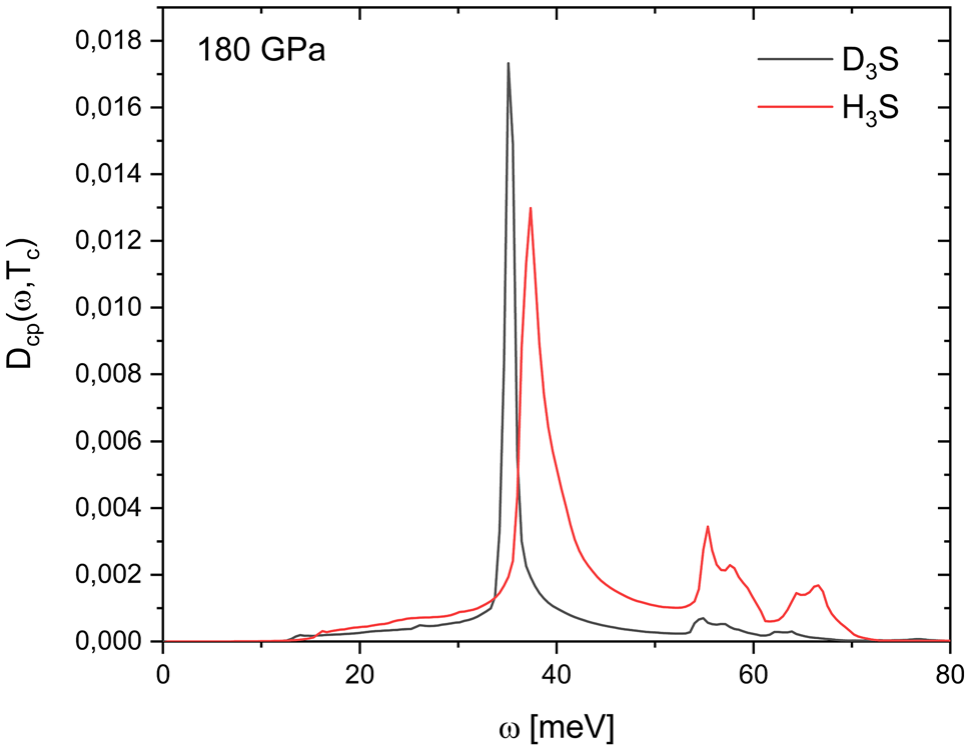
Comparison between Cooper-pair distribution functions $D_{cp}(\omega ,T_c)$ of $\hbox {D}_3\hbox {S}$ (black line) and $\hbox {H}_3\hbox {S}$ (red line) calculated at 180 GPa.

In order to extend this analysis, in Fig. 3 the comparison between the $D_{cp}(\omega ,T_c)$ of $\hbox {D}_3\hbox {S}$ and $\hbox {H}_3\hbox {S}$ calculated at 180 GPa is presented. The comparison between the $D_{cp}(\omega ,T_c)$ of $\hbox {D}_3\hbox {S}$ and $\hbox {H}_3\hbox {S}$ allows for a direct analysis of these systems. It is observed in Fig. 3 that the $D_{cp}(\omega ,T_c)$ functions bear some similarity between them, although the $D_{cp}(\omega ,T_c)$ of $\hbox {H}_3\hbox {S}$ shows the contribution of two small peaks at 55 and 65 meV. The intensities of $\omega _{cp}$ in these functions show an appreciable difference, being greater for that of $\hbox {D}_3\hbox {S}$, however that of $\hbox {H}_3\hbox {S}$ is slightly centered 2.25 meV above the other one. The most important difference is observed in the area under the function, which means a higher value of $\hbox {N}_{{cp}}$ of $\hbox {H}_3\hbox {S}$ (see Table 2).

**Table 2:** Superconductor properties of $\hbox {D}_3\hbox {S}$ and $\hbox {H}_3\hbox {S}$ calculated at 180 GPa.

	$\hbox {T}_c$ [K]^4^	$\hbox {E}_F$ [eV]	$\lambda$	$\omega _{cp}$ [meV]	$\hbox {N}_{{cp}}$ ($\times 10^{-3}$)
$\hbox {D}_3\hbox {S}$	150.4	16.9461	2.4815	35.10	48.53
$\hbox {H}_3\hbox {S}$	185.6	16.9463	2.4774	37.35	100.19

Some superconductor properties ($\hbox {T}_c$, $\hbox {E}_F$ and $\lambda$) of $\hbox {D}_3\hbox {S}$ and $\hbox {H}_3\hbox {S}$, including $\omega _{cp}$ and $\hbox {N}_{{cp}}$ are presented in Table 2. It is observed that $\hbox {E}_F$ and $\lambda$ are almost identical values, and the $\omega _{cp}$ values differ only by 2.25 meV. The most significant difference is observed between the $\hbox {N}_{{cp}}$ values, them being proportional to experimental $\hbox {T}_c$ values (a high $\hbox {N}_{{cp}}$ implies a high $\hbox {T}_c$). $D_{cp}(\omega ,T_c)$ function allows to establish qualitative differences in conventional superconductors through the $\hbox {N}_{{cp}}$ parameter, which shows proportionality with $\hbox {T}_c$.

### Sulfur isotope effect in $\hbox {H}_3\hbox {S}$

**Figure 4:**
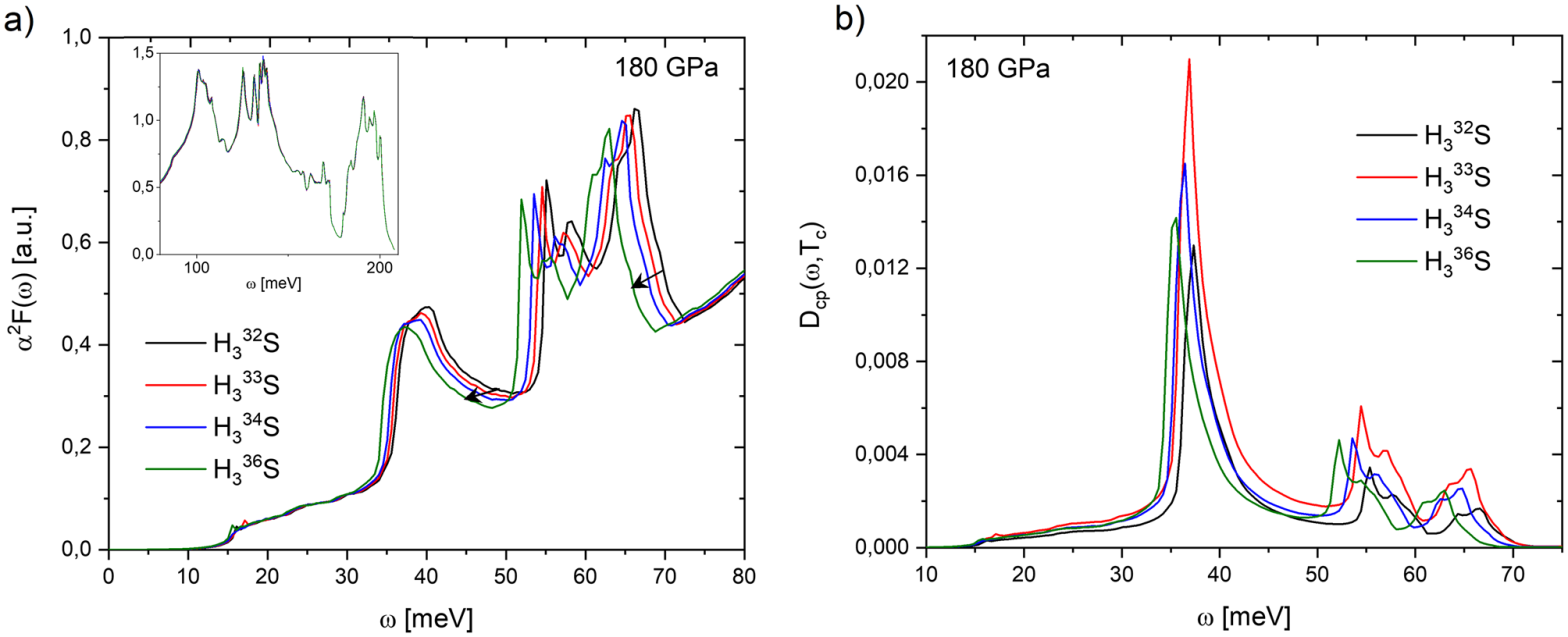
(**a**) Eliashberg spectral $\alpha ^2F(\omega )$ and (**b**) $D_{cp}(\omega ,T_c)$ functions of $\hbox {H}_3^{32}\hbox {S}$, $\hbox {H}_3^{33}\hbox {S}$, $\hbox {H}_3^{34}\hbox {S}$ and $\hbox {H}_3^{36}\hbox {S}$ calculated at 180 GPa (arrows show as Eliashberg function smooths as isotope mass increases). The inset in (**a**) presents the $\alpha ^2F(\omega )$ function from 80 to 220 meV. Note only changes in $\alpha ^2F(\omega )$ are observed in the energy range of 10–70 meV.

Eliashberg spectral $\alpha ^2F(\omega )$ and $D_{cp}(\omega ,T_c)$ functions of $\hbox {H}_3^{32}\hbox {S}$, $\hbox {H}_3^{33}\hbox {S}$, $\hbox {H}_3^{34}\hbox {S}$ and $\hbox {H}_3^{36}\hbox {S}$ at 180 GPa are shown in Fig. 4.

It is observed in Fig. 4a that the substitution of $^{32}\hbox {S}$ atoms by the heavier isotopes $^{33}\hbox {S}$, $^{34}\hbox {S}$, and $^{36}\hbox {S}$ has effects mainly on $\alpha ^2F(\omega )$ function in the energy range of 30–70 meV, thus generating a shift of the spectrum towards lower energies as the mass of the isotope increases. This result is due to the fact that the S atoms contribute with vibrational states only in this range of energies^13^. What is significant here is the fact that these small spectral variations induce important changes in the $\hbox {T}_c$ (see Table 3), that is S atoms play a relevant role in the high $\hbox {T}_c$ of $\hbox {H}_3\hbox {S}$ (as in $\hbox {D}_3\hbox {S}$).

**Table 3:** Comparison between theoretical $\hbox {T}_c$ values reported by Szczesniak^13^, and $\omega _{cp}$ and $\hbox {N}_{{cp}}$ values obtained from $D_{cp}(\omega ,T_c)$ functions calculated for $\hbox {H}_3^{32}\hbox {S}$, $\hbox {H}_3^{33}\hbox {S}$, $\hbox {H}_3^{34}\hbox {S}$ and $\hbox {H}_3^{36}\hbox {S}$ at 180 GPa.

$\hbox {H}_3^{n}S$	$\hbox {T}_c$ [K]	$\omega _{cp}$ [meV]	$\hbox {N}_{{cp}}$ ($\times 10^{-3}$)
$\hbox {H}_3^{32}\hbox {S}$	193.63	37.35	100.19
$\hbox {H}_3^{33}\hbox {S}$	209.00	36.90	164.23
$\hbox {H}_3^{34}\hbox {S}$	199.20	36.45	127.51
$\hbox {H}_3^{36}\hbox {S}$	193.91	35.55	114.82

The $D_{cp}(\omega ,T_c)$ functions of $\hbox {H}_3\hbox {S}$ calculated with the influence of the substitution of stable S isotopes (see Fig. 4b) show that there is no change in the range of Cooper pairs formation energies (10–70 meV). However, an increase in the intensities of the peaks $\omega _{cp}$ is observed with respect to the calculation for $\hbox {H}_3^{32}S$, being higher for $\hbox {H}_3^{33}S$. These results lead to changes in the $\hbox {N}_{{cp}}$ values. The comparison between theoretical $\hbox {T}_c$ values reported by Szczesniak^13^, and $\omega _{cp}$ and $\hbox {N}_{{cp}}$ values obtained from $D_{cp}(\omega ,T_c)$ functions for stable S isotopes is presented in Table 3.

It is observed from Table 3 that there is again a direct correlation between $\hbox {N}_{{cp}}$ and $\hbox {T}_c$ values. An $\hbox {N}_{{cp}}$ of 164.23$\times 10^{-3}$ implies a $\hbox {T}_c$ of 209 K, the highest $\hbox {T}_c$ reported for the substitution of $^{32}\hbox {S}$ atoms by the isotopes $^{33}\hbox {S}$ in $\hbox {H}_3\hbox {S}$ at 180 GPa^13^. While for the characteristic peak $\omega _{cp}$, the substitution of S in $\hbox {H}_3\hbox {S}$ by stable isotopes of S induces a small variation of 1.8 meV, due to the shift toward lower energies of the $D_{cp}(\omega ,T_c)$ function. These results show that the change of S isotope mass has an influence on the superconducting state of $\hbox {H}_3\hbox {S}$, confirming its classical electron–phonon interaction.

Our results show that the low-vibration region, indicates by $D_{cp}(\omega ,T_c)$ function, has an important role in the superconductivity, which is confirmed by the fact that a simple variation in the low-frequency region leads to important changes in $\hbox {T}_c$.

## Conclusions

In this work, we present the analysis of Cooper-pair distribution functions $D_{cp}(\omega ,T_c)$ of the compressed $\hbox {D}_3\hbox {S}$ and $\hbox {H}_3\hbox {S}$ high-$\hbox {T}_c$ conventional superconductors. The effect of all stable sulfur in $\hbox {H}_3\hbox {S}$ was calculated as well.

For all systems studied, the $D_{cp}(\omega ,T_c)$ function calculated showed that the Cooper pairs formation energy is located at the energy range 10–70 meV (low-frequencies). From each function was calculated the $\hbox {N}_{{cp}}$ parameter, which is proportional to the total number of Cooper pairs formed at temperature $T_c$. All cases revealed a correlation between the $\hbox {N}_{{cp}}$ parameter and experimental (or theoretical) $\hbox {T}_c$ value reported. A high $\hbox {N}_{{cp}}$ implies a high $\hbox {T}_c$. This suggests that $D_{cp}(\omega ,T_c)$ function is a theoretical tool that allows establishing an initial qualitative characterization of conventional superconductors (through the $\hbox {N}_{{cp}}$ parameter), which is not possible from the direct analysis of Eliashberg spectral function $\alpha ^2F(\omega )$ and Phonon density of states (PhDOS).

Although it is expected that hydrogen atoms be protagonist in the superconductor properties, the $D_{cp}(\omega ,T_c)$ obtained from substitution of $^{32}\hbox {S}$ atoms by the heavier isotopes $^{33}\hbox {S}$, $^{34}\hbox {S}$, and $^{36}\hbox {S}$ reveal that S atoms play a relevant role in the high-$\hbox {T}_c$ of $\hbox {H}_3\hbox {S}$.

Finally, $D_{cp}(\omega ,T_c)$ function showed again that the low-vibration region is where Cooper pairs are possible, which indicates the importance of this region on the electron–phonon superconductivity. This has been confirmed by the fact that a simple variation in the low-frequency region can lead to important changes in $\hbox {T}_c$.
